# The Hospital. Nursing Section

**Published:** 1903-08-29

**Authors:** 


### The Hospital.

**Nursing Section.**

Contributions for this Section of "The Hospital" should be addressed to the Editor, "The Hospital" Nursing Section, 28 & 29 Southampton Street, Strand, London, W.C.

No. 883.—Vol. XXXIV. *SATURDAY, AUGUST* 29, 1903.

### Notes on News from the Nursing World.

### LORD SALISBURY'S NURSES.

During the illness of Lord Salisbury, which terminated fatally on Saturday evening, the illustrious patient was attended by three nurses, one of whom was always at hand. The value of the services of trained nurses has long been recognised by members of the family at Hatfield, and every provision was made for them as soon as the medical advisers of Lord Salisbury considered it essential that the number should be increased to three.

### THE INDIAN NURSING SERVICE.

Last week we published the revised regulations for Queen Alexandra's Military Nursing Service for India, and as so many nurses desire in these days to extend their experience to India, it may be worth while to add that each candidate for an appointment, unless she was born in India, must send in a certificate of birth—a certificate of baptism will be accepted in the latter case—must state the profession or occupation of her father, whether she is single, married, or widow, and supply a medical certificate as to the condition of her health. She must give as references the names of the matron and of the two senior medical officers in the hospital at which she was trained; a list of appointments held since the completion of her training; and the names and addresses of two ladies to whom reference may be made as to social position. This information is embodied in the formal declaration signed by the candidate, and on her appointment the declaration is stamped as an agreement according to law.

### PREVENTABLE POVERTY.

The Victoria Trained Nurses' Association have determined to establish a benevolent fund to benefit the poorer sisters who have "fallen by the way," and the honorary secretary is appealing to all members of the nursing profession in the Australian colony to assist to their utmost. She contends that a really good nurse, who has good health, should not only be able to secure a sufficient safeguard from poverty by joining the Royal National Pension Fund for Nurses, but can also afford to give a small subscription each year to help others. She therefore proposes that every nurse earning over £50 per annum should subscribe 10s. 6d. yearly to the benevolent fund, those earning less 5s., 2s. 6d., or even 1s., according to their means; and goes on to urge that none of the members can divest themselves of a certain amount of responsibility with regard to the less fortunate of their fellow workers. The benevolent fund has just assisted a nurse of the Alfred Hospital who became incapacitated from further work by ill health, the former and present members of the nursing staff—and others—having subscribed £310 to provide her with a pension of 10s. a week. The surplus, after paying this amount, is to go to the general fund to make loans or gifts to other members in distress or sickness. The movement, it will be seen, is quite outside the Royal National Pension Fund, as a benevolent fund cannot be administered from afar: the Junius S. Morgan Benevolent Fund, which does so much for the Pension Fund nurses at home, cannot extend its ministrations further afield. The honorary secretary of the Victoria Benevolent Fund says: "Rich and influential people in England have done a great deal to help nurses to secure a pension, and English nurses who are not so well paid as nurses in Australia have joined in thousands, while here they have not joined in tens," and adds as a reason why they should do so that "there is positive disgrace in preventable poverty."

### THE METROPOLITAN COMMON POOR FUND.

A complaint was lately made to the Camberwell Guardians that better accommodation for the nurses at the workhouse infirmary was badly needed. When the nurses are in possession of their new quarters, which will be ready for occupation early next month, it will be impossible to say that they are not adequately provided for. The account given in another page by our Commissioner of the accommodation set apart for them in the new Camberwell Infirmary shows that the Guardians desire to keep pace with the times, and to afford the nurses in their employment every reasonable comfort. We regret that their wish to secure an adequate staff cannot, however, be realised at present. As the medical superintendent informs our Commissioner, the Metropolitan Common Poor Fund blocks the way. The salaries of the staff nurses are a charge upon that fund, and so long as an infirmary is within the Metropolitan area, the number of nurses has to be regulated, not by the requirements of the patients, but by a cast-iron rule. This is absurd, and whatever objections may be urged to the suggestion of Mr. Keats, that there should be a general rate for the whole of England, the managers of the great London Poor-law infirmaries should be able to appoint the number of staff nurses they require.

### THE MEMORIAL OF THE WORKHOUSE MASTERS AND MATRONS.

In the memorial of the Association of Workhouse Masters and Matrons, which has been sent to the President of the Local Government Board, efforts are made to prove that it is not desirable that the superintendent nurse should have the supreme control which is recommended by the Departmental Committee on Workhouse Nursing. It is contended that the merits of superintendent nurses as a class still remain untried, and where tried individually as administrators the trial has not proved to be worth the risk necessarily attending the transition from one system to another. The masters and matrons therefore submit that "until it is demonstrated that superintendent nurses have superior administrative ability to themselves, the constitutional method of procedure is to leave the old order of things unchanged unless it be admitted that the time has now arrived when changes ought to be made for the sake of making changes." The answer to this would seem to be that as the old order of things urgently stands in need of amendment, Mr. Long will be fully justified in giving to superintendent nurses the trial as a class which has so far been denied to them. They, at any rate, have not, like the untrained matrons, been weighed in the balance and found wanting.

### MUSIC AND DISTRICT NURSING.

The visitors at Looe, one of the prettiest little watering-places in South Cornwall, must be congratulated upon the manner in which they have come to the aid of the District Nursing Association. There happened to be among them several musical artists of note, who consented to give their services at a concert on behalf of the association, with the result that on Thursday last week the Guildhall was quite inadequate to accommodate all who wished to attend. At the close of the entertainment, Lady Trelawny, vice-president of the organisation, thanked the visitors at Looe for the musical treat the audience had enjoyed, and for the substantial assistance offered to the funds.

### NURSES AND VACCINATION.

In view of the fact that small-pox is prevalent in Newcastle-on-Tyne, a number of hospital nurses have made a statement through the local newspapers for the purpose of warning persons who apply to the magistrates for certificates for exemption from vaccination of the risks they run. They are hopeful that some who will not listen to the advice of doctors may pay heed to the appeals of nurses; and, speaking from practical experience, they affirm not only that they find that those small-pox patients who have never been vaccinated have the disease in a much more aggravated form than those patients who have been vaccinated 40 or 50 years before, but also that the unvaccinated class of patient is dreaded by nurses on account of the loathsome way in which the disease shows itself. This dread has, of course, to be overcome, but it need never be entertained if people would take advantage of the preventative of vaccination, which enables nurses to feel perfectly secure against personal danger of catching small-pox.

### DISTRICT NURSING AT DARLINGTON.

The second annual report of the Darlington District Nursing Institute is in several respects a satisfactory document. It shows an excellent record. During the year the nurses paid upwards of 6,000 visits, and attended 362 cases; and though the income was smaller than in the preceding year, this, it is explained, was due to the fact that when the organisation was started several subscribers paid two years' subscription in advance. There is a balance of £244 in hand, of which £100 is placed on a deposit account at the bank. Perhaps the most encouraging feature of all is that the contributions of the working men have increased to the extent of more than £20. The efforts of the committee to induce the works in the town to subscribe in their corporate capacity, have also met with some response.

### NURSING HOMES IN GLASGOW.

An interesting correspondence is going on in the Glasgow press respecting the establishment of nursing homes for patients of the middle classes who can only afford to pay a small weekly sum, varying from half a guinea to fifty shillings a week. Conflicting opinions are expressed as to whether the need would be better met by starting new institutions or by developing those which are already existing. We notice that it is contended that to put such a suggestion into practical form, "some of the leading wealthy citizens would require to provide the capital sum needed to open a home free of debt." This is all very well, so long as those who find the capital sum receive an undertaking that as soon as the home pays its way they will be entitled to interest on their money. From the outset the scheme, as it is foreshadowed by its advocates, should be conducted upon business principles. First it seems to us the co-operation of the hospitals should be sought.

### A CHAMPION OF RETROGRESSION.

At a meeting of the Chichester Board of Guardians, Mr. Butler proposed that instead of having "outside help" in the infirmary the Board should revive the system of pauper nursing. The chairman promptly met this proposal with the rejoinder that he could never go back to pauper nursing, and the Hon. Mrs. Grosvenor observed that the inmates were miserable under the former system. We are glad to say that Mr. Butler did not find a single supporter of his retrogressive views, but it is a matter for regret that there should still be left guardians of the poor who wish to deprive them, when they are sick and suffering, of the attention to which they are entitled by Act of Parliament, as well as on the grounds of common humanity.

### IN THE INTERESTS OF HEALTH.

At a meeting of the Sheffield Board of Guardians exception was taken to the proposal of the Hospital Extension Committee for the erection of a covered way between the Infirmary and the Nurses' Home. The objection was that the corridor "was not needed," but we are glad to see that it was not successfully maintained. In winter and in wet weather the advantages of a covered way between a nurses' home and the main building ought to be obvious to everyone. In fact, it is so much a necessity in the interests of health that whenever a new nurses' home, apart from the hospital, is erected, it should, as a matter of course, form part of the scheme.

### SHORT ITEMS.

An organ recital at the Music Hall, Barnstaple, last week, in aid of the Nursing Fund, realised a considerable sum.

### The Nursing Outlook.

"From magnanimity, all fear above;
From nobler recompense, above applause,
Which owes to man's short outlook all its charm."

### THE VICTORIAN ORDER OF NURSES.

There are some women whose special gift lies in seeing what is needed, and in starting new movements, or aiding those that are in their early days of struggle. This is peculiarly true of the Countess of Aberdeen, whose name is held dear in Canada for many good works, but among nurses chiefly for the founding of the Victorian Order of Nurses.

The movement began as a memorial of the Diamond Jubilee in 1897, and aimed at supplying hospital trained nurses to those dwellers in the Dominion who could not afford the 10 or 15 dollars a week that are the wage of the private nurse in Canada. Nurses were very rare six years ago, even in the cities of Montreal and Quebec, and in the outlying districts they were practically unknown. Yet it required a good deal of skill and perseverance to get the Victorian Order started and to make it popular: an unstable woman might well have failed in the work, but Lady Aberdeen, with her motto of "Blessed be Drudgery," saw the thing through, and now the Order is one of the institutions of which all nurses are proud. The charter of the Order was granted at Windsor on May 1st, 1898, and permission was given to the members to wear a similar uniform and a similar badge (save for the wording) to that of the Queen's nurses in the United Kingdom. This forms a pleasant bond with our cousins beyond the sea, and the fact that Lady Aberdeen is now resident in this country, and always ready to help the nursing movement here, cements the feeling of fellowship.

But the Canadian nurse has tales to tell which no Queen's nurse on this side can equal: for at the time of the opening up of the Klondike region in 1898 four nurses were sent up, partly by the Government, partly by special funds. The last 150 miles which Nurse Powell undertook with the advance guard of soldiers was all heavy marching, while on arrival at Dawson an appalling work awaited her. Typhoid was at its height, and two log buildings were made into a hospital to hold 36 patients. But what a hospital! The ground was covered with shavings to keep out the damp, there were few beds and still fewer mattresses; often a patient lay for days with only a blanket between him and the boards. There were no disinfectants, no dressings, no towels, and only two or three cups and plates. No wonder that after four weeks Nurse Powell herself got typhoid and was seriously ill, but returned to work again in Dawson as soon as possible. And other nurses have done equally difficult pioneer work, for one of the objects of the Fund is to start small cottage hospitals where they are badly needed, and then to send up a nurse in charge. So in the big, beautiful, but lonely regions of the great North-West Territory the trained nurse is penetrating, and seems to find work and a hearty welcome. There are now more than twenty branches of the order, and more than 50 members at work, but Miss Macleod, the superintendent, reports that one of the great difficulties is to get an adequate supply of suitable probationers. The nurse of to-day is apt to find the country dull after her training in a city hospital, and she does not like the outposts of civilisation. And every probationer must hold a good certificate of general training, and then has to undergo four months of special drilling in district work either at Montreal or Toronto, then she signs on for two years and has to go wherever she is sent. She is provided with a complete outfit and paid 300 dollars a year, with maintenance and laundry. To the woman who loves a healthy and adventurous life, who has got some of the Robert Louis Stevenson spirit in her, and is not a mere pavement-trotter and haunter of shop windows, to enter the Victorian Order is to find an outlet for all that is best. Exactly as the Queen's nurse here holds a position of peculiar honour and certitude, so does the Victorian nurse in Canada. She is known and respected everywhere; and she always has the force of a great movement behind her to help her to make her way in difficult places. At present Lady Minto is interesting herself very much in the extension of the Order, particularly in the opening of cottage hospitals and then using them for the training of Indian girls. But the sad news from Canada is that after these five years of hard toil in organising and training Miss Charlotte Macleod, chief lady superintendent, is about to resign. Miss Macleod is a Canadian from New Brunswick, and was head of the Waltham Training School for many years. Her name, however, will always be associated with the Victorian Order of Nurses, to whose success she has so largely contributed, and all the members of which wish her a pleasant rest after her labours. It was suggested when the Order was started that it would come into conflict with the private nurses, with the Sisters of Charity, who did district nursing in Quebec and elsewhere, and also with those hospitals, such as the Western Hospital, Montreal, which sent out nurses to certain poor patients. Time has proved these fears to be vain; but the smooth working is undoubtedly due to the personal courtesy and charm of the founder of the Order and of the first superintendent. We sincerely hope that in the future the Order may be as fortunate in its friends and officers as it has been in the past, and we feel sure that the influence of the Countess of Aberdeen and of Miss Macleod will still be at the service of the society for which they have done so much.

### Lectures on Medical and Surgical Nursing.

By John Hopkins, F.R.C.S., Medical Superintendent of the Central London Sick Asylum District Asylums, Cleveland Street, W., and Hendon, N.W.

### LECTURE XV.—INJURIES.

Contusions or bruises are at times of such importance that a nurse should acquire the habit of noting and reporting any that she sees. When a part is pinched or crushed, there is a rupture of blood-vessels under the skin, and blood is effused into the cellular tissues. This gives rise perhaps to obvious swelling, the stretched skin glistening over it. After a few days the colouring matter of the blood is carried nearer the surface and gives the skin a dull purplish colour, which then becomes somewhat rusty in tint, and last of all of a greenish yellow. These changes in colour are due to alterations taking place in the blood pigment preparatory to its absorption. A valuable inference might be drawn from the probable date of a bruise. Sometimes blisters rise over the bruised part. They are most frequently seen in fracture of the leg.

Blisters are more frequently seen as the result of friction, generally on the feet. If the blister be broken, there is an excoriation. An abrasion is due to the cuticle being rubbed off. However slight the breach of surface may be, it is worth attention, for though in the healthy it will generally give rise to no trouble, in the unhealthy it may lead to serious results.

Wounds are divided into clean-cut wounds, and others in which the edges are bruised or torn. An incised wound is more easily cleaned than the others, and is more likely to heal up without suppuration or by first intention, as it is called. When grit and dirt have been ground into the tissues, the part thus affected must suppurate and perhaps will slough away. When wounds heal by first intention, the cut surfaces adhere together, and in a few days are so firmly united that it would require a little force to separate them. Stitches can generally be removed on the fifth day. All that it is necessary to do in such cases is to watch the temperature, which is generally a sure indication as to the condition of the wound. If it remains normal, the wound is aseptic and healing. When a wound heals by suppuration, there is a growth of granulations on the raw surface, and the wound is filled up by these being superimposed. The wound heals from the bottom. If by any chance the edges of it adhere together and enclose pus within, then the temperature goes up, for absorption of poisonous products of the suppuration takes place. Hence this must be avoided and the wound is plugged with gauze or other dressing, or a drain of some kind is passed to the bottom of it. Care has to be taken that pus does not become enclosed. When there is suppuration the edges of the wound are red and swollen; they are inflamed, and this inflammation may be very severe.

Hæmorrhage from wounds may be arterial, venous or capillary. The manner of stopping the bleeding will depend upon what kind of vessels the blood is coming from, so it is necessary to be able to recognise which kind of bleeding is taking place. When arteries bleed, the blood is spirted out a considerable distance if the end of the vessel is exposed, and the colour of the blood is bright red. Venous bleeding is in a continuous non-pulsating stream. When the blood is coming from the capillaries there is simply a general oozing.

Arrest of bleeding from the capillaries is easily effected by pressure, either by pressing the sides of the wound together or by putting direct pressure upon the cut surface. Venous bleeding is easily arrested by elevating the part, or by applying direct pressure, or by compressing the vein beyond the wound. As the blood is flowing in the veins from the toes to the heart the pressure must be below the wound.

Bleeding from arteries is sometimes easily stopped by direct pressure upon the bleeding point, but if the wound be a deep one and has been inflicted with a puncturing instrument, pressure on the wound will be of no use. Then you must put pressure on the main trunk that supplies the limb. This is readily done with the fingers and thumb grasping the limb firmly over the arterial trunk; but you cannot keep this pressure up very long, and you cannot always be certain of compressing the artery fully, so some more effectual means is necessary. This you always have at hand. A band tied round the limb above the wound and at a point where there are no bony prominences to prevent the artery being compressed, is an effectual way, and this can be provided in a moment, for as a last resort it is always possible to tear a strip from one's own clothing. Simply to tie the band round the limb is not enough. Knot it loosely so that you can pass your hand through it, then pass a stick, a ruler, a poker, anything at hand through it and twist this round until the band is screwed up tight and until the bleeding stops.

Secondary hæmorrhage, once of very great importance, is not often seen now. It occurs after hæmorrhage has been arrested by ligature of the arteries or other means. Owing to some failure in the process of healing, the ligature gives way and the artery begins to bleed again. Where this is to be feared, careful watch for the first signs of bleeding is required, and the means for arresting the hæmorrhage, a tourniquêt, dressings, and everything required for opening up and closing the wound again must be at hand.

Fractures of the bones are classified as simple and compound. The fracture is compound where the broken bone has penetrated through the skin and forms an opening by which dirt with its bacteria can gain entrance. Where the bone is broken into several fragments it is said to be comminuted. The repair of a fracture is effected by means of a material that grows from the surface of the bone beneath the periosteum near the fracture, and from the surface of the fracture itself. This substance is called soft callus, and it forms a mass which envelops the broken ends of the bone, holding them together. It eventually hardens by being converted into bone, and is then spoken of as hard callus. Before the soft callus forms there is a period of a few days, during which effused blood is absorbed. A knowledge of these points in the repair of a broken bone is of value. One knows that it is possible to readjust the splints with advantage during a certain number of days, after which it is not expedient to attempt the correction of any displacement.

When a bone is broken the soft tissues are much damaged near the break, blood is extravasated, and there is often much swelling from the effusion of serum from the blood also. This takes place some hours after the accident, and if the splints are put on before the swelling takes place, it may be found that the circulation is arrested and gangrene may result. Hence it is necessary to keep a close eye for 24 hours upon all fresh fractures recently put up, and it is sometimes found necessary to take off and re-apply the splints. In preparing the splints let the pad be firm but not hard. The pad is put on to prevent the hard splint from causing pressure sore, and it must be just firm enough to effect this object. Presssure of the bandage also will cause sores, so spread ointment is required to protect and keep sweet any point of likely pressure. When the thigh-bone is broken it is necessary to keep the body and the broken limb of the patient on one plane, otherwise the body of the patient sinks in the bed, the splint becomes uncomfortable, and the fragments are disturbed. In order to prevent this pieces of board are placed on the bedstead under the bed. These are called fracture-boards.

Fracture of the spine is likely to be accompanied by paraplegia, retention of urine, and cystitis. Bed-sores are peculiarly liable to form of the very worst kind and to prevent this a full-length water-bed should be used. In attending to such a case care is required to avoid putting any strain upon the spine when moving the patient.

Fracture of the skull is often associated with concussion of the brain, hæmorrhage upon the brain, and coma or inflammation. Operations for hæmorrhage or for removing fragments of bone that press upon the brain may be necessary. The trephine is used to remove a circular piece of bone in order to get at the damaged part. In preparing for the operation the scalp must be shaved and thoroughly cleaned.

Injuries of nerves may cause loss of sensation and motion. Sometimes the injury leads to neuritis, which makes the part supplied by the injured nerve peculiarly tender and the greatest care is needed in handling it. Patients who use crutches may get crutch paralysis through the nerves in the axillæ being pressed upon. When a part has lost sensation or when the patient is insensitive through loss of consciousness hot-water bottles must be used with the greatest caution. Not only should they be wrapped up in flannel, but they should never be put in contact with the patient.

Dislocations call for little attention when once reduced. Stiffness of the joint follows and this is removed by rubbing and passive movement, but it must be done with caution lest the dislocation recurs, as it is sometimes apt to do.

### Milk and its Dangers.

BY A NURSE.

On reading the interesting article on "Fever in the Milk-can," published in The Hospital some time ago, I am tempted to send you a few remarks on the subject of milk as an article of diet for children over the age of infancy (12 months) and for grown-up people.

There can be no doubt that the mother's milk is the best food for all infants, and if they get a sufficient supply from a healthy mother the best foundation for a strong and vigorous adult life is effectually laid. It is this natural food for infancy that has maintained the life and vigour of the countless millions of India, China, Japan, and Russia, in spite of poverty, famine, plague, pestilence, floods, wars, and the horribly unsanitary conditions under which those peoples live.

But the question arises "Ought milk to form so large a portion of the food of children and adults as it does in England at the present time?"

It is hard indeed to fight against popular opinion, but I wish to place before your readers a few facts for consideration.

There has been of late years a very large increase in the consumption of milk by people of all ages in this country. Have we benefited by it? Are our people stronger, better grown, more capable of resisting disease, and have they better teeth than formerly, or than those numerous nations which never use milk as food after the age of infancy?

Certainly the Chinese and Japanese never use milk after infancy, and yet I have read of the marvellous feats of endurance those people can perform. Recently a German officer reporting on some Japanese troops stated that large bodies of their men marched 70 miles per day, each carrying a load of 80 lbs., and he stated that no European troops could make such an incredible march. The Chinese coolies perform marvellous feats in carrying weights over enormous distances, their only food being rice and weak tea. In Northern Italy and in Chile the quarrymen and miners work most vigorously for many hours every day on polenta and water, their wages being about one shilling per day, but milk forms no part of their food. I could, but will not now, multiply instances of this kind, but will merely mention that at least some of the English farmers, men of education and intelligence, do not believe in the value of milk as food for their children or adults, but simply laugh at those who think it is so valuable. But those gentlemen are liberal with food such as eggs, bacon, fowls, meat, and soup, porridge, fruit, etc.

Now there are other points to be considered:—1st. Milk can be so easily contaminated and can carry disease more readily than any other fluid. 2nd. It is easily affected by the weather. 3rd. It is in many cases very difficult of digestion. 4th. When other descriptions of food are taken with it they frequently cause certain changes to take place in the milk which cause biliousness and many stomach disorders besides very severe headache, bad temper, and a host of other evils.

Against this must be placed the fact that those peoples, such as the Scotch, who lived almost exclusively on oatmeal and milk, and the Irish, who lived on potatoes and milk, were more healthy and stronger than they have been since they have taken to tea, tinned meats, and bread. But then their diet in those old days used to be absolutely simple, whereas our diet in these days is extremely complex. Formerly sugar was very little used; now we consume five times the quantity we consumed 60 years ago, and this may in a measure account for our being unable to digest milk as the labouring classes did in former days.

Now there is an enormous demand for milk, and it is probable that this great demand has led to a forced production of milk from high feeding, etc., which has deteriorated the quality of the milk. Then in many houses it is almost impossible to keep the milk, even for a few hours, without running great risks of infection or contamination from sources too numerous to mention.

I wish only to open the door for discussion of these unpopular views, so I will conclude by making a few suggestions which I hope may be carefully criticised. When there is any possibility of doubt about the purity or contamination of the milk after its reception, would it not be well to substitute broth, beef-tea, or soup for milk? Would it not be possible to substitute thoroughly well-cooked porridge with bovril, gravy, broth, syrup or jams, for the ordinary porridge and milk?

I have known many elderly people who cannot take milk or cream without suffering severely, and who certainly do not derive any benefit from it, either when in health or when recovering from disease. Speaking from recent personal experience during my recovery from a very severe illness, I may say I gave up milk and took beef-tea, broth, etc., with marked and immediate benefit. I admit the benefit of milk in some stages of disease, but I doubt its suitability in many cases for growing children and adults. I do not presume to lay down the law in this matter, but I would ask, Have we not made milk too much of a fetish of late years?

### The Nurses of Camberwell Infirmary.

BY OUR COMMISSIONER.

Early in September the patients in Camberwell Workhouse Infirmary will be removed into the wards of the new buildings in Brunswick Square, and the nurses will enter into possession of their new quarters. When I called to see over the latter and to obtain some full information as to conditions under which the nursing is carried on in an institution intended to provide for the needs of a parish containing nearly 300,000 souls, with a very large proportion of poor and necessitous people, the matron, Miss Frances Marquardt, was away, but the medical superintendent most courteously offered to show me over the infirmary and to answer any questions as to the training of the probationers, the work of the nurses generally, and the provisions which have been made for the comfort and well-being of the staff.

"Well, to begin with," I said, "what is the number of the nursing staff?"

"Up to now it has been, with two male nurses, 60; but in the future, when the old part of the infirmary has been renovated and made equal to the new, and the number of beds is augmented to 800, the nursing staff will consist of the matron, two assistant matrons (one of whom is home sister), two night superintendents, 15 sisters, 45 staff nurses and 55 probationers. The staff nurses hold a three years' certificate."

### The Common Poor Fund.

"Will that be adequate, supposing that the 800 beds are occupied?"

"No, we should like to have 45 staff nurses, which would mean a fully-trained nurse on every floor by day and night. But I suppose that we shall have to do with 25. Owing to the salaries of the staff nurses being a charge upon the Metropolitan Common Poor Fund, the Local Government Board set a limit on the number they allow to each infirmary. I may say that the Common Poor Fund allow a fixed sum to each Metropolitan Union or parish for the medical staff, including the dispenser—who can only be paid a high salary with the result of the medical officer receiving a lower one—and irrespective of whether three medical officers are needed, or five. The same with the nursing staff. If an increase in the staff is asked for, the authorities of the Common Poor Law Fund plead in refusing that there is no necessity for any more nurses, as the staff is already equal to that of other institutions of similar size, quite regardless as to whether there are acute or chronic cases, whether any amount of pauper help is given, or any special needs of the institution. The allowance of officers is strictly according to the rateable value of the union or parish. If we were outside the Metropolis, we could have as many nurses as we required."

### Beds Always Full.

"But cannot such a state of things be remedied?"

"We shall be very glad if The Hospital can help us to get it remedied. To make clear the necessity for a staff nurse to each floor of wards by day and night, I may say that of 36 cases on each floor the great majority would be acute. We are always discharging cases, and always full. In fact, we have to get rid of patients before they are well. The medical and other inspectors of the Local Government Board are entirely in favour of an adequate staff, the Guardians desire it, and the Common Poor Fund is the only barrier that stands in the way. So far as I can see, there is no other remedy but to get a general rate for the whole of England.'

"That, of course, is a big question?"

"Yes, it is one that would have to be included in a scheme of Poor Law Reform, of course. Every rate-payer should be able to avail himself of infirmary treatment and pay only according to his means. As it is, however, our nursing staff is proportionately the largest of any Poor Law infirmary within the Metropolitan area."

### The Course of Training.

"How long has the period of training for probationers been three years?"

"The school was formed four years and a half ago under Miss Marquardt, the present matron. Miss Marquardt was trained at the Royal Free Hospital. She was afterwards sister at the Poplar Hospital for Accidents, matron of the General Hospital at Barbadoes for a year, night superintendent at Greenwich Workhouse Infirmary for four years and a half, superintendent of nurses at Birkenhead Workhouse Infirmary, where she organised a training school. Her experience has, therefore, been very varied. No matron could be more just to the nurses, nor more interested in their welfare than Miss Marquardt. Her one aim is to make them happy. She is very firm, but very devoted to them; and she is a splendid organiser."

"Does the matron lecture?"

"Both she and myself give lectures during the whole of the three years. We each teach what we consider essential. She has, naturally, more to say about nursing, and I more about medicine and surgery. During their training the probationers are given opportunities of attending operations, and they also take duty in male, female, children, and infants' wards; they are also allowed to go into the infectious blocks. In the lectures they are instructed as to the preparation of meals and dietary generally. At the end of the training they have a course of lectures on sick cookery. This is after they receive their certificate, but they always stay on for the sick cookery course."

"If we have a vacancy for a sister," continued the medical superintendent, "it is awarded to the most capable of the nurses who have completed their training. One half of our sisters are of outside training and the other half of our own. The guardians, who are very enthusiastic and helpful and try to do all they can to promote the happiness of the nurses, have hitherto accepted the recommendations of the matron and myself."

### Salaries and Age.

"Do the guardians appoint the probationers?"

"It is their prerogative to make appointments at the end of three months' trial, but if they find that both the matron and myself recommend an appointment they always sanction it. The three months' trial are included in the period of training. Applications are made to the matron, who interviews applicants, and after obtaining satisfactory references reports the result. The present salaries are £10 the first year, with uniform, £12 the second, and £14 the third, with £4 a year in lieu of beer. Travelling expenses are paid by the guardians to candidates who are desired to attend."

"What is the age limit?"

"From 21 to 30. The matron always has a number of suitable candidates to select from. When a fresh selection has to be made we advertise; but we get many candidates who are recommended to apply by nurses in course of training."

### Hours on and off Duty.

"Are the hours long?"

"On the contrary, they are unusually short. The staff-nurses and probationers breakfast at 6 30 A.M. and are on

### THE NURSES OF CAMBERWELL INFIRMARY—*Continued.*

duty at 7. They have twenty minutes off in the middle of the morning for lunch and to make their beds, three-quarters of an hour for dinner, half an hour for tea, and they are off duty at 8.30. They also get two hours of the day off duty, and once a week, instead of the two hours, they are free from 6 until 10 P.M. On Sunday they are off in the morning, afternoon, and evening alternately, and then, if the cases permit, they have always an opportunity of attending a place of worship. Once a month they have one whole day and one half day off duty, and through the kindness of the matron they are allowed to have breakfast in bed the day they are off duty. This is much appreciated. They go to bed at 10, and lights are out at 10.30. Every nurse has annually a holiday of three weeks."

"And the hours of the night nurses and sisters?"

"The night nurses are on duty from 8.30 P.M. to 8 A.M. The hours are so arranged that the day and night nurses work together in the morning for an hour with the most serious cases at a time when the ward work is heaviest. Sisters come on duty at 8, after breakfast at 7.30, and go off duty at 9 P.M. They go to bed at 10.30. Otherwise there are no differences. In addition to the regular off-duty time, all the members of the staff have a liberal allowance of late leave."

### Varying Uniform.

"I see that the uniforms are not the same."

"No. The sisters wear navy blue, the staff nurses a blue of a different shade, and the probationers blue and white. We like all the nurses to wear uniform when they are off duty for two hours, but not for longer periods. They are expected to provide their own outdoor uniform. One feature in connection with the nursing staff is their unity. They are like one big family. I attribute this entirely to the matron, who never listens to tattle or gossip of any kind."

"How many wards are there in the infectious block?"

"Seven, and the number of staff nurses in the block varies according to the number of cases. Although we have no lying-in wards, we take in urgent midwifery cases, and during their general training the probationers have lectures given them on midwifery. I explain the instruments used."

### The Nurses' Home.

"Now about the nurses' quarters?"

"In the old quarters, which they are about to vacate, there was a separate cubicle for every nurse. But in the new Home each has a bedroom of her own. The whole of the probationers sleep in the Home which, in addition to four bedrooms on the ground floor, and 40 on each of three floors, contains a large general recreation-room, a dining-room for the nurses, a general reading and writing-room, a sitting-room for the sisters, a recreation-room for the probationers, and a quiet room where no talking is allowed. The assistant matron, who is also home sister, is in charge of the Home, and has her own sitting-room in the building."

"You have not mentioned bath-rooms."

"There is one bath-room to every ten nurses; also two large box-rooms on every floor. All the bedrooms are furnished alike in birch, and contain wardrobe with panel glass of full length, washstand with a green-tiled back and marble top, dressing chest and drawers with a bevelled plate looking-glass, and hanging cupboards of pitch pine in which ornaments or books can be kept. The floor of each room is cement covered by linoleum, and all rooms are warmed by hot-water coil and have a small flue in connection with the electric fan, the building being lighted by electricity. There is an excellent system of ventilation, and the plan of heating by warm water saves all the dust of coal fires."

### Sisters' Accommodation.

"The sisters, I gather, will not sleep in the home?"

"No; only the staff nurses, the probationers, and the domestic servants, who, by the way, have their own sitting and recreation-rooms. The bedrooms of the sisters, and also the matron's rooms are in the front portion of the administration block, but the meals of all the staff are taken in the Home. The bedrooms of the sisters are much the same, both as to size and furniture, as those of the staff nurses and probationers."

### Recreation and Diet.

"There remains only, I think, the question of recreation."

"The guardians give me the grounds to manage for the staff, and in suitable weather there is tennis, croquet, and bowls. We have also an asphalte tennis court for use at any time of the year. Indoors the principal recreation is dancing, and there are the ordinary games of healthy girls. The nurses have always an hour after supper for recreation or reading. They have their own library which is managed by themselves."

"I should like to add," concluded the medical superintendent, "that the guardians are very liberal in their allowance to the steward for the nurses, and that the diet is exceptionally good. There are always two joints of meat at least on the dinner-table, and if the matron finds she has too much bread or meat, she can exchange it for fruit, or eggs, or anything she chooses. There is no waste, but there is considerable variety of diet, as well as abundance; and to this ample and excellent diet I largely attribute the good health which is enjoyed by all the members of our nursing staff."

Mr. Keats then took me over the whole of the new buildings, including the spacious and finely-appointed kitchen, and the block for children, which is in itself a perfectly-equipped hospital. Both the nurses' Home and the quarters for the sisters in the administrative block are furnished with admirable taste, and while comfort has evidently been anxiously considered, there has been no indulgence in superfluous luxury.

### TRAVEL NOTES AND QUERIES.

Route to Johannesburg (Nurse E.).—The cheapest way is *viâ* Durban, second class, £24 10s. 1d. I am sending you by post a paper of the Natal Line prices and routes. You will see a steamer carrying second-class passengers leaves on Sept. 4th. Many only take first-class so you must be careful as to dates. You will see at the foot of the paper the rules concerning baggage. You can take anything you like by paying for it, but the bulk of luggage goes into the hold. No amount of money will allow of your taking more than the regulation cabin trunk with you in your berth. Apply to the steamship company for the dimensions allowed; all companies have not the same regulations. With regard to the expense of living in Johannesburg, I cannot answer with any accuracy, because at present and for some months the town is in a transition state in consequence of the late war and ensuing circumstances. It will be for some time a little expensive, more so than England, but that condition will not last.

A Holiday in Sicily (A. P.).—I hope you will see this, but you give no pseudonym. Sicily is not a cheap place to travel in, and you could not see all the chief places of interest, but it is a lovely country, and I am sure you will enjoy it. This is my idea. Go first to Taormina from Syracuse where you will land (Syracuse is not interesting, but to stay the night or perhaps two write to Villa Politi for rooms). At Taormina go to the Grand Hôtel Belle Vue; terms from 6 francs 50 cents. A good view of Etna from here. There is plenty to see at Taormina to occupy a week. Then on to Palermo, about 55 miles. This is the gem of Sicily. Write and ask terms for rooms on third or fourth floor to the Hôtel Trinacria and Hôtel de France. Return *viâ* Catania. If you think this too extended a tour and therefore too expensive you must leave out Palermo, a visit to which will add at least one-third to the cost of your holiday, but then it is the place of all to be seen.

### Nurses and Dispensing.

### LONDON SCHOOLS OF PHARMACY.

Dispensing as an occupation for women is so much upon the increase, and questions as to the cost of instruction, the locality of schools, and the necessary time for study to ensure success, are so frequently being sent to us that we consider it advisable in the interest of many nurses to publish details of the respective schools. It will be seen that the oldest of the London schools was founded as long as 30 years ago, when chemists were always of the male sex, and the idea that women could learn to dispense so well and so correctly as men had not dawned upon the world of self-supporting workers. But since then a great advance has been made, and now not only are special lecture halls with special class-rooms and special dispensaries provided for the use of ladies in one institution, but scholarships are thrown open to both men and women. In one instance a college of pharmacy has been started where none but women are prepared for examination. As the number of positions at hospitals and infirmaries which may be filled by women increases, so will the total of lady dispensers ready and eager for work. As it is a profession which yields fairly pleasant, fairly healthy, and fairly remunerative employment this is a matter for congratulation. The following particulars have been personally obtained from the heads of the various institutions named, who are, of course, exclusively responsible for the information given.

### THE IMPERIAL COLLEGE OF CHEMISTRY AND PHARMACY.

Day and evening classes are held at 49 and 51 Imperial Buildings, Ludgate Circus, by Mr. Frederick Davis, member of the Society of Public Analysts. Mr. Davis removed to his present rooms in 1896 from Newington Causeway, where he had been since January 1888. Personal and individual instruction and supervision is the method used by Mr. Davis, who prepares for the examinations of the Pharmaceutical Society, Apothecaries' Hall, the Institute of Chemistry, and the Conjoint Board. Fresh courses are begun in January, April, July and October. The fees are, for the Pharmaceutical Course (three months), £10 10s.; six months, £15 15s. Single subjects may be taken, the fees being as follows: Chemistry, £3 3s.; Botany, £2 2s.; Materia Medica, £2 2s.; Pharmacy, £2 2s.; Prescriptions, £1 1s.; Practical Chemistry, according to arrangement. Students must provide themselves with test tubes, platinum foil and wire, charcoal and blowpipe. The fees are otherwise inclusive. "Students are taken exclusively by Mr. Davis, and every effort is made by him to impart the requisite knowledge in such a manner that its lasting and sound character may be of paramount value to the student in after life. During term students visit places of pharmaceutical and chemical interest, under the direction of the principal, such as the Import Drug Warehouses of the London Docks; the Geological Museum, Jermyn Street, W.; the Science Collections, South Kensington; the Royal Botanic Gardens, Regent's Park; gas-works, and chemical factories, etc." Lectures are delivered in one room, illustrated by means of blackboard, diagrams, specimens, etc., while in the other practical work is carried on. The lectures are followed by questions and tutorial work; "such a method," says Mr. Davis, "not only ascertains the progress of the student, but gives that facility of expression which so many candidates lack when under examination. The theory followed minutely with practical work, and so going hand in hand, the student gains a sound and thorough knowledge of the subjects impossible to obtain by any other means. Cram is strenuously avoided." Ladies are admitted. Mr. Davis had a good deal of experience as a practical chemist before he turned his energies to teaching.

### MR. H. J. CARSON.

Women who wish to qualify for passing the Assistants' Examination of the Society of Apothecaries of London may be prepared by Mr. Henry J. Carson, at 7 Great Castle Street, Oxford Circus. This is neither a school nor a college, for Mr. Carson, who has had some years' experience as a master in secondary schools in the colonies, describes himself as a private coach.

The preparation lasts six months, since the Board of Examiners for the Certificate—Practical Pharmacy make six months' instruction imperative. The teaching for this examination, as well as for the minor and major examination of the Pharmaceutical Society, is individual and personal. There are no lectures or classes, and the laboratory and the dispensary are distinct and separate. Students, either men or women, may enter at any time, and if from any cause the course is interrupted, the work may be resumed later, and the fees already paid will be credited. This concession has been made to meet the case of nurses and others who are preparing for examination while carrying on the duties of their profession. Students must attend for three hours every day but Saturday, this may be either morning or afternoon. No home work is given, but if a student is anxious to succeed (and they are, Mr. Carson says, "exceedingly keen") she naturally continues her work at home. Sometimes, if a student is obliged to begin earning almost immediately, the work is so arranged that she shall be qualified to give assistance to a dispenser—though it must be distinctly understood that she is only partially trained—after three months' preparation; she can then continue her studies as a half-timer.

The whole of the work—practical and theoretical—is gone through twice in six months, four examinations being held in the year.

Tuition Fees are as follows: £1 11s. 6d. per month, and £8 8s. to the October Examination. (N.B.—This inclusive Fee is the same whether the Student commences work at once or on April 14th, the last day of entry here for the October Examination.) It has just been arranged that a Class shall be held two evenings a week, Tuesdays and Thursdays, from 7 P.M. to 9.30 P.M., commencing March 3rd, 1903. The Fee is £1 1s. per month. This class is only available for those who cannot attend during the day. No previous knowledge of Latin or Chemistry is required.

There are usually about nine ladies at work, some being nurses, some girls fresh from school, and some older women. Although a good general education is very desirable, Mr. Carson does not consider it indispensable; he, however, states that arithmetic is frequently a weak point, "the students can work a sum easily enough, but they do not seem able to think in percentages, and decimals frighten them."

Mr. Carson informs us that all his students have secured comfortable posts after qualification. He has been training ladies for dispensing for about four years.

Scientific examinations are Mr. Carson's speciality, and men are received for coaching in the evening.

(*To be continued.*)

### Preparation for Colonial Nursing.

BY A NURSE IN THE GOLDEN WEST.

As a nurse of 20 years' experience in England and the United States—Kansas, Iowa, and California—I was much interested in reading in the *Nursing Section* of The Hospital the account of the initiation of a colonial college to teach the practical side of life to those (especially nurses) contemplating taking up work in a foreign country. Nothing I can write could give anything like an idea of what an inestimable benefit such a training would be to them. When I came to the United States 15 years ago, I thought that after seven and a half years' training in English hospitals, I was well fitted to cope with any nursing which might fall to my lot, but I was soon undeceived.

### Managing the Cows.

One of my first cases was a family, seven in all, down with typhoid fever, living on the prairie 25 miles from the nearest doctor. He only came once, for the parents, though ill themselves, refused to allow him to prescribe alcoholic stimulants and other treatment, so he left in disgust, and I was sent to "do my best" for them and their sick children. When I arrived on the scene, I found my only assistants were a good-hearted old "coloured lady," who as a rule took in washing, did cooking, and was afflicted with a deformed hip, and a cowboy who came in every other day to do the rougher outdoor work, and who as regularly for the first three weeks made me an offer of his hand and heart every time he came. There were pigs, horses, chickens, and cattle to feed—and two cows to milk. Those cows, specially one named "Muley," I can never forget. I could fool every other being on the ranch, even to administering the forbidden stimulants disguised, and disflavoured by coffee, quinine, etc., and getting my patients to swallow calomel by calling it simply by its Latin name. The driving horse, a true Western broncho, I managed to catch by strategy in the shape of a can of oats. But those cows could not be tricked. They knew I was of the weaker sex each time and took a mean advantage. I went so far as to don masculine attire one day when the youngest boy was crying for warm new milk, and advanced with as bold a front, and as manly a whistle as I could command, swinging my tin pail in the jaunty fashion my cowboy admirer did. But even then, when with aching fingers I had squeezed out about a quart of the precious fluid from Muley's swollen udder, she suddenly elevated her heels and sent me and the bucket somewhere, where there were a great many stars appearing before my sight. Aunt Maudy, the "coloured lady" aforementioned called to me "Honey, try agin; youse done got on de wrong side ob de ol' Bossy;" but I did not take her advice, as I could see nothing of the "bossy" but her hind legs and swishing tail as she sailed out on the prairie triumphantly, and left me with my nose bleeding profusely.

### A Nurse as Grave-Digger.

Soon after I had sent my cowboy admirer away for four days by telling him very decidedly that his attentions were unwelcome, the mother, down with the fever, gave birth to a still-born baby. I had to make its little coffin out of old boxes, by myself—Aunt Maudy having a horror, like many negresses, of anyone dead—and put her to watch the others meanwhile. Then indeed I realised how much harder it was to use a saw, hammer, nails, etc., than to watch a carpenter do the same, and wished I had helped my small brothers in early days. I almost fear that if my cowboy friend had appeared on the scene when the saw had cut a jagged wound in my hand and I had brought down the hammer more than once on to my own nails instead of the steel ones, that I should have willingly promised to adorn his shanty for the rest of my days as his "old woman" if he would have finished the job for me. As it was I had to do it all by myself, and then to dig so deep that the coyotes could not get at the body, and after that, at the request of the poor mother, sing a hymn over it. She chose several which I had never heard of, but at last we compromised on "Onward Christian Soldiers," she being lifted on to a cot near the window so as to take part in the ceremony. This was no easy matter for I was about fifty feet away, and her voice being weak and slow, I was generally a few words ahead. Just as the family were beginning to mend, Aunt Maudy had to leave, as her daughter was about to increase the coloured population, and this left me with the cooking, etc., on my hands.

### Cleaning the Chickens.

When I took out of the oven my first batch of bread (I can laugh now, but then I was too tired, and not in a "Mark Tapley" frame of mind) intended as my mainstay for a few days, it was about as hard and heavy as a two-year-old Dutch cheese. The chickens which Aunt Maudy before leaving had kindly beheaded for me I had laboriously plucked, and it being my first experience, I had pulled off with the feathers nearly all the skin, so that they looked as if they were suffering from some disease. But that was not all. After pulling out their insides, I put them on to boil, and about half an hour after, remembering that cookery books said "remove the scum which will be found floating on the top," I took off the lid. To my horror, not only scum but a medley of grass and grains of all kinds was visible on the surface, and I realised that I had forgotten to empty and clean their crops! I must acknowledge that I sat down in despair and fairly cried; in the middle of which my cowboy assistant and a comrade of his arrived!

### The American Cowboy.

For nature's gentleman commend me to the American cowboy. They may shoot each other on sight, chew, spit, and swear, but when it comes to seeing a woman in distress they will lay aside everything to give her a helping hand. In this case the two young fellows rolled up their sleeves, and in less than an hour the table was set. On it was a dish of chicken stew, with the loveliest hot dumplings, and the mealiest potatoes, light rolls, and some tempting chicken soup for the sick ones. After the meal was ended they turned to, washed the dishes, and gave a general clean-up, and next morning brought over a buxom old dame to take over the cooking and washing, from whom I learned much which helped me in after cases. This is only a trifling sketch, and it would be easy to write a volume on the difficulties which a colonial nurse may be called on to encounter at any moment. Nurses who contemplate roughing it, whether on "Greenland's icy mountains or India's coral strand," the "Golden West," or "Afric's sunny plains," should determine to be jack of all trades, and learn to milk and to carpenter, to mend shoes, to cook, harness a horse, ride, drive, and in fact how to do all manner of rough work. They will never regret it.

### Echoes from the Outside World.

### Movements of Royalty.

Last week the King, who is still at Marienbad, attended a dinner given in his honour by Sir Francis Plunkett. It is stated that in obedience to his Majesty's wish, the ménu was in accordance with the cure regulations. The King entered his name in the visitors' book with the other guests. Now that his Majesty's new motor car has arrived he is able to take long expeditions to Kuttenplau and Plau, to Karlsbad and elsewhere, which naturally increases his enjoyment. The King also sent Lord Cranborne—now Lord Salisbury—a message of condolence as follows:—"Accept my deepest sympathy, and express the same to your brothers and sisters on the occasion of the loss of your distinguished and ever-to-be-regretted father.—E. R."

### Death of Lord Salisbury.

The nation mourns the death of the Marquis of Salisbury, which occurred at Hatfield on Saturday night at ten minutes past nine. Lord Salisbury had been out of health for some months, but it was not until a fortnight ago that his illness assumed a serious aspect and caused special anxiety to his friends. Lord Edward Cecil, who was on his way to Egypt to rejoin the Sirdar's staff, could not be reached in time for him to return to say farewell to his father; but the rest of the family, including the Prime Minister, were present when he passed away. Lord Salisbury, who was born on Feb. 13th, 1830, in the house where he died, was the second son of the second marquis, and first made his name in the world of politics and literature as Lord Robert Cecil. On the death of his elder brother in 1865 he became Lord Cranborne, and in 1868 he succeeded his father in the title and family honours. As Lord Cranborne he held the post of Secretary for India, and as Lord Salisbury he was in succession Secretary for India, Foreign Secretary, and three times Prime Minister of England. On Feb. 26th, 1901, he reached the record of long service among modern Prime Ministers, and on July 11th, 1902, he tendered his resignation to King Edward. In the *Court Circular* of Sunday night his Majesty paid the following tribute to the memory of the illustrious statesman:—"The King has received with profound regret the news of the death of the Marquis of Salisbury, and deeply deplores the loss of so great a statesman, whose invaluable services to Queen Victoria, to the King, and his country in the highest offices of the State which he held for so many years will ever dwell in the memory of his fellow-countrymen." The interment will take place at Hatfield on Monday, and a memorial service will be held on the same day in Westminster Abbey at two o'clock.

### The Balkan Crisis.

It was announced on Monday that, in consequence of the prompt satisfaction given by Turkey to the demands made by Russia in respect to the murder of M. Rostkovsky, the Russian squadron has been ordered to return to Sebastopol. The Porte, with unusual expedition, sent its reply to the Russian Embassy 12 hours after the presentation of the Russian Note. Meanwhile the rebellion is extending, and according to news from Adrianople, the insurgents have destroyed telegraphic communication at several points between Adrianople and Kirk Kilise, and have burned two villages because the inhabitants refused to join in the insurrection. The British warships *Ramillies* and *Vulcan* are in the waters of the Turkish Archipelago, ready to proceed to Salonica in case of need.

### Fighting in Northern Nigeria.

On Friday it was announced that the Colonial Office had received a telegram from the Government of Northern Nigeria announcing that on July 27th a successful attack was made on the town of Burmi, which was completely destroyed, after severe fighting. The ex-Sultan of Sokoto and most of his chiefs, with some 700 of their followers, were killed. The British loss was one officer—Major Marsh, who served in operations on the Niger in 1897-98, and also took part in the South African war in the defence of Mafeking, being twice mentioned in despatches—four rank and file, and six carriers killed, as well as three British officers, 62 rank and file, and seven carriers wounded. The main force is advancing in Fika, but no further fighting is expected, and the operations are practically concluded.

### Cardinal Vaughan's Successor.

The new Archbishop of Westminster, in succession to the late Cardinal Vaughan, was chosen on Monday by the Congregation of the Propaganda in the person of the Right Rev. Francis Bourne, the well-known Roman Bishop of Southwark. Dr. Bourne was born at Clapham on March 23rd, 1861, and is therefore only 42 years of age. He was educated at St. Cuthbert's College, Ushaw; St. Edmund's, Ware; St. Salpice, Paris; and the University of Louvain. Ordained Priest in 1884, Dr. Bourne became rector of Southwark Diocesan Seminary in 1889, Coadjutor to the Bishop of Southwark in 1896, and Bishop in 1897.

### The Yacht Race.

The first race for the *America* Cup between Sir Thomas Lipton's *Shamrock III.*, the challenger, and *Reliance*, the defender, commenced on Friday last week, but owing to the bad weather it was officially declared "off" at 3.40 p.m. On Saturday morning the contest was concluded, with the result that *Reliance* won by 7 minutes 2 seconds. The race is admitted by Sir Thomas Lipton to have been a fair test of the sailing abilities of the two boats. The wind mostly held true, and though it became puffy occasionally towards the close, *Shamrock* had as much, if not more, benefit than the American boat. The second race, which was held in ideal weather, resulted also in the victory of *Reliance* by only 1 minute 19 seconds. The wind dropped at the finish of the race, which was unfavourable to the challenger. A storm arose after the race, and for a short time *Shamrock III.* was in danger.

### The Humbert Frauds.

The Humbert case came to a termination on Saturday, and Madame Therese Humbert made her long-promised statement. She declared that the Crawfords did exist, but said that they called themselves by the name of Regnier. These people, she asserted, amassed a great fortune in 1870, through purchasing French Rentes at the panic prices which prevailed during the war. M. Labori stated that Regnier was the intermediary between Marshal Bazaine and Prince Bismarck at the surrender of Metz, and that he was condemned to death for treason. The jury, who had to decide 258 questions, found all the prisoners guilty, and the judge sentenced Madame Humbert and her husband to five years' solitary imprisonment, Romain D'Aurignac to three years', and Emile D'Aurignac to two years' imprisonment. The sentence imposed upon the convicts is generally regarded as exceedingly light.

### The Nurses' Bookshelf.

Pocket-Handbook for Monthly Nurses. By M. Chenery. (Simpkin Marshall. 1s. net.)

Here is a handy little manual which will be invaluable to maternity nurses. Its directions are concise, practical, and full of helpful suggestions. It is clearly printed, and in its bijou form will be easily carried in the nurse's bag. The authoress modestly disclaims any originality in its compilation. "It has been published in response to frequent requests for information on the small yet important duties of a nurse in the lying-in room, and for suggestions as to feeding the mother and infant. The information has been gained from lectures at Queen Charlotte's Hospital and from personal experience."

How Mary Fitzgerald Learned Housekeeping. A Domestic Economy Reader for Irish Schools. By Charlotte O'Conor Eccles. (Eblana Press, Dublin: Fallon and Co., Limited. Price 8d.)

Miss Charlotte O'Conor Eccles, as a fellow-worker with Sir Horace Plunkett in his strenuous efforts to improve the condition of his nation, is peculiarly well qualified to write such a book as this little primer of domestic economy, in which under the guise of a story, many useful instructions are given in the first principles of housewifery. Many of the recipes and nearly all the advice is as suitable for English homes as for Irish ones, but there are points—for example the objections to earthen floors, and the suggestion as to thatching a roof with heather—which would not have occurred to a writer who did not know personally the conditions of Irish peasant life. The cookery instructions also are based on a like familiarity with the potentialities of wages and their purchasing power in the sister isle. The book, however, deals only with country life, where a garden and a few fowls may be kept by all. We hope Miss O'Conor Eccles will write a second volume adapted for town children. For a girls' reader in country schools this is admirable.

Our Baby: for Mothers and Nurses. By Mrs. J. Langton Hewer, Certif. Obstetrical Society, London; late Hospital Ward Sister. Eighth edition, revised. Thirtieth thousand. (Bristol: John Wright and Co. London: Simpkin Marshall, Hamilton, Kent and Co., Limited. 1902. Price 1s. 6d. paper, 2s. 6d. cloth.)

The success of Mrs. Langton Hewer's little book is clearly shown by the fact that it has reached its eighth edition. It is a very thorough and practical little manual, totally free from the mawkish sentimentality which used to be considered correct in books dealing with maternity and babyhood, and it contains a great deal of really useful information. We regret to note that the author occasionally drops into the habit of recommending the goods of special manufacturers. This is a fault that clings to all books of this class. In itself it is trifling, but some young mothers are so anxious-minded that they think their infants will not thrive unless they can procure just the food or the clothing mentioned in the manual on which they pin their faith. This is, however, a slight fault, and one which we could have completely forgiven Mrs. Hewer if she had named among the firms to whom she gives this gratuitous advertisement even one who would provide the sensible little shirts worn by American babies, made of fine wool or silk and wool, quite open in front, and fastening like a child's stays by long strips passing under the arm and being fastened with a safety pin at the back. These are easily put on, and form a comfortable double covering for the lungs and abdomen. But, so far as we know, they cannot be obtained in this country.

### Everybody's Opinion.

[Correspondence on all subjects is invited, but we cannot in any way be responsible for the opinions expressed by our correspondents. No communication can be entertained if the name and address of the correspondent are not given as a guarantee of good faith, but not necessarily for publication. All correspondents should write on one side of the paper only.]

### NURSES' UNIFORM.

"A Man" writes: Hospital nurses need not worry if their uniform be sometimes worn by those who have no right to wear it. Imitation is the sincerest form of compliment after all. The average man, moreover, does not mistake the nursemaid with a perambulator for the hospital worker; and his admiration for the latter is not for the comeliness of her costume but for those qualities of courage, unselfishness and devotion of which her uniform is the badge. Without those qualities in the wearer, of which her occupation is the guarantee, her dress of itself has no attractions in the eyes of men; and the latter can gauge more accurately than may be generally supposed between the golden article and the imitation dross.

"Maud Ruel," a trained nurse, writes: Judging from the tone of both Miss Eleanor Jarvis's and Mr. Frederick Jayne's letters in this week's Hospital, I do not think that anyone could ever make them understand that it would be bad taste to wear nursing dress at a wedding or ball. The full dress uniform of both naval and army officers is quite a different matter. Personally, I hope to see the day when only district nurses and private nurses need to wear the uniform of their calling. It is absolutely necessary with the former, and decidedly useful to the latter. But even then it is nice to wear a neat coat and skirt with suitable hat—all from the same tailor, as many patients, especially gentlemen and boys, do not like driving or walking with a nurse in uniform. I cannot see the real need of any nurse in hospital being compelled to wear a distinctive dress out of doors, and it has the effect of causing so many women to forget how to dress, as they cannot often afford uniform and private costumes.

### THE ONE-YEAR NURSE.

"Three-Year Trained" writes: Having trained for three years in one of the very excellent training schools mentioned in the letter of "An Infirmary Nurse" in your issue of the 8th inst., I beg to say a few words in favour of the "three-years' nurse." However "capable" and "intelligent" a nurse may be, I fail to see how she is to obtain an intimate knowledge of the various symptoms of the many diseases she will be called upon to nurse. I consider that observation cannot be cultivated in so short a period. Let it be understood that the training schools mentioned train for three years, and if such an adequate knowledge can be obtained in 12 months, what is the object of training for three years? I also maintain that the best qualities a nurse can produce will be as much required in nursing the sick poor as in any other department of the profession. I think too that the "thoroughly in earnest," "capable," and intelligent nurse will not be satisfied with 12 months' experience. The longer a nurse is in the profession the more important this becomes. I found that at the end of three years my term of training had been only too short to become thoroughly acquainted with the work in the many departments that a large training school affords. With regard to salary, the "One-Year Nurse" cannot complain, for she is often to be found working side by side with the three-year nurse, and frequently in a more elevated position.

### NUNS AS NURSES.

"A Trained Nurse" writes: Mr. Wyndham's answer to Mr. Farrell on the subject of the "Granard Nursing Scandal," given in the House of Commons, forms a curious pendant to "Cecilia's" letter in last week's Hospital. He says the nuns "do not care to perform, and ought not to be asked to perform, some parts of hospital work, and therefore it was essential that they should have the assistance of trained nurses" Cecilia argues that "nuns and nurses should be criticised on the same grounds, *i.e.* professionally." Very well, any nurse who "did not care to perform" and "ought not to be asked to perform" disagreeable nursing duties would not be allowed to remain in a hospital, and would certainly be told she had mistaken her vocation. That the "hospital work" which the nuns "did not care to perform" was part of a nurse's duty we know, because as the nuns refused to do it "it was essential to engage trained nurses to assist them" by doing the work they despised. So much for the "professional" aspect; and now for the moral point of view. "Cecilia" claims that "nuns are the only body of nurses" for whom "the simple rule of 'all for Thy sake' is the very keynote of their existence," and she brands trained nurses as "one of the most irreligious bodies of women." But it was the professedly self-sacrificing and devoted nuns who "did not care to perform" unpleasant work, and it was to the members "of one of the most irreligious bodies of women" that the patients owed the care they received when the nuns deserted them. Is there none, in these days, who "thanks God that he is not like other men are," but when put to the proof "passes by on the other side," like the Levite of old, leaving distasteful services to be performed for the sick and suffering by the Samaritan whom he despises? "By their fruits ye shall know them."

### Appointments.

[No charge is made for announcements under this head, and we are always glad to receive, and publish, appointments. The information to insure accuracy should be sent from the nurses themselves, and we cannot undertake to correct official announcements which may happen to be inaccurate. It is essential that in all cases the school of training should be given.]

Axminster Cottage Hospital.—Miss Rosa M. Ford has been appointed sister. She was trained at the Guest Hospital, Dudley, and the West London Hospital, Hammersmith, W.

Bath Statutory Hospital.—Miss Crofts has been appointed matron. She was trained at the Bristol Royal Infirmary, where she has since been sister of medical wards and temporary night sister. Miss Crofts holds the L.O.S. certificate.

Fulham Infirmary.—Miss Jessie F. Ballantyne has been appointed matron. She was trained at High Barnet Cottage Hospital, and Guy's Hospital, London. She has since been sister, night superintendent, and assistant matron at Lewisham Infirmary, and matron at the Incorporation Infirmary, Shirley Warren, Southampton.

Gateshead Hospital for Infectious Diseases.—Miss Jane Brand has been appointed matron. She was trained at the London Hospital, Whitechapel, and has since been senior charge nurse and deputy matron at Middlesbrough Sanatorium.

General Hospital, Singapore and Straits Settlement.—Miss Alice Smith has been appointed sister. She was trained at the General Infirmary, Leeds, and has since acted as sister of the women's medical and surgical wards at the Hull Royal Infirmary. She holds the L.O.S. certificate.

Home for Aged Mariners, Egremont.—Miss Edith Muriel Ardern has been appointed charge nurse. She was trained at the Birkenhead Union Infirmary, and was sister at the same institution during twelve months. She has since acted as assistant matron at the St. Leonard's Convalescent Home, and as temporary charge nurse at the Clayton Infirmary.

Leeds Union Infirmary.—Miss Mabel Ethel Maughan has been appointed charge nurse. She was trained at the Mile End Infirmary, London, E, where she was afterwards charge nurse. She has also been engaged in private nursing at Bournemouth.

Passmore Edwards Hospital, East Ham.—Miss S. Finn has been appointed staff nurse. She was trained at St. Olave's Infirmary, Rotherhithe.

Queen Alexandra's Royal Naval Nursing Service.—Miss M. M. Harlock has been appointed sister. She was trained at the Seamen's Hospital, Greenwich, and has since been night sister at the Walsall and District Hospital.

St. Mary's Hospital, Manchester.—Miss A. K. Brooks has been appointed matron. She was trained at the Royal Infirmary, Hull, where she was afterwards sister during four years, and night superintendent during one year. For the last four years she has held the post of assistant matron at St. Mary's Hospital, Manchester.

Samaritan Hospital for Women, Liverpool.—Miss E. Maude Halliwell has been appointed matron. She was trained at the Royal Infirmary, Newcastle-on-Tyne, afterwards undertaking private nursing for a short time. She acted as a sister of the Army Nursing Reserve in South Africa in 1900 to 1902, and has since held temporary posts at the above hospital.

Stanmore Cottage Hospital.—Miss Eva Goring has been appointed staff nurse. She was trained at the Lowestoft Hospital, and acted as staff nurse at the Norfolk and Norwich Hospital, and at the Victoria Hospital, Northwich. She also undertook temporary duty at the Ilkeston Hospital.

### Presentations.

The Gordon Hospital, Vauxhall Bridge Road, S.W. A pleasant little ceremony was enacted at the Gordon Hospital for Fistula, etc., on Thursday afternoon last week, the occasion being a presentation to the Matron, Miss Maxwell, who is retiring after having held this arduous and responsible position for the last 10 years. Patients, past and present, had joined in subscribing for, and selecting as a parting gift, a very handsome clock in an oak case, with a suitable inscription at the foot. The clock was accompanied by a testimonial, beautifully printed in gold, signed by all the donors, the kind thought and gift of one of the patients.

The presentation was made in the men's ward by the Secretary of the hospital, Mr. H. Rede Buckley, who made a touching reference to the high esteem in which Miss Maxwell was held by all who knew her, and to the deep regret which was felt in the hospital at her departure. The ward was crowded with patients, past and present, together with some of the Matron's old friends, and presented quite a bright and festive appearance. The nurses and probationers of the hospital had previously given the Matron a beautiful wrought-iron and copper lamp, while the Secretary gave her a silver-handled bread fork.

Miss M. E. Moir, one of the head nurses, who is leaving the hospital to take up work in foreign parts, was also made the recipient of a handsome travelling rug, of the Gordon tartan, as a mark of the high esteem and regard in which she is held. This, too, was subscribed for by the patients, with whom Nurse Moir is deservedly a universal favourite, and was accompanied by an illuminated card with the names of the donors inscribed on it, the work of a recent patient.

Altogether, the scene afforded a striking evidence of the love and esteem which grows up amongst the matron, nurses, and patients in a London hospital.

### For Reading to the Sick.

"WE LIFT OUR HEARTS TO THEE."

How many thousands are worshipping now!
The Lord looks down where His loved ones bow!
Solemn and sweet are the strains that rise
From the haunts of earth to the holy skies.

Where the tall cathedral rears its dome,
The long, loud notes of the organ roam
Through Gothic arches, and nave, and aisle,
Where the last red beams of the sunlight smile.

Bright Angels hover where childhood sings,
And the first faint prayer of the contrite springs;
And they gather the soft low words that come
Where the household kneel by the hearth of home.

But the curtains of night the landscape shade;
And the voices of earth in silence fade:
There's a Land where Life hath no shade nor care—
How many thousands are worshipping there!

*Charles Lawrence Ford.*

"Surely goodness and mercy shall follow me all the days of my life: and I will dwell in the house of the Lord for ever.' At once these words, "goodness and mercy," attract our attention. It was "goodness and mercy" that led us first out of the fold, with an aim and object in life. There was "goodness and mercy" in that shelter from the noontide heat. But now it is "goodness and mercy" all the days of my life. And we think of grace which is not only preventing and accompanying, but also subsequent. We owe a great deal to the grace that comes after, the grace that follows us; the grace that not only gives us the wish to do what is right, not only the grace that starts us and helps us in what is right, but also the grace which helps us to finish.

Make your life all temple, all part of the sacred enclosure. Enlarge it towards the east, where we look for our Saviour's coming. Let it be a life of patient witness for God. Enlarge it towards the west, where the sun of our life is gently dipping towards the grave, in a life of preparedness. "I will dwell in the house of the Lord for ever." This should be our aim, to attain to the realisation of the life hid with Christ in God; and to this God is separating us off, that our sojourn with Him may be eternal.—*Canon Newbolt.*

When we lie on our own bed of sickness, we shall not regret having reached out a helping hand to others who have little or no alleviation of their pains, and the act will assuredly not be forgotten by Him Who hangs upon the Cross to-day, and Who said, "Whosoever shall give to drink a cup of cold water only in the name of a disciple, verily I say unto you, he shall in no wise lose his reward."—*Anon.*

Sing softly all day in your heart
Glad praises to your Guest adored,
And let this sweet thought cheer your way,—
"Thy Presence dwells within me, Lord."

And let the light of His dear Love
Shine forth in all your words and ways,
Beaming on others, for His Sake,
And helping them to love and praise.

*Anon.*

### Notes and Queries.

FOR REGULATIONS SEE PAGE 257.

*Uniform.*

(228) Will you be good enough to tell me if I can wear a grey uniform? I am a trained maternity nurse, but am now working privately; and I greatly prefer grey, to the usual blue, uniform.—*C. J.*

You may wear any coloured uniform you like.

*Hospital Training.*

(229) I am a lover of nursing, strong, healthy, and 23 years of age. I have, however, only had a Board School education, and I should be glad to know of a London hospital which would accept me.—*B. K.*

Write first to the Lady Superintendent, the Middlesex Hospital, Mortimer Street, W. If you fail, consult "The Nursing Profession: How and Where to Train" in the choice of a school. There is no reason why you should not be accepted at many London hospitals.

*Nursing Consumption.*

(230) I should be glad to know how I could get a post in an open-air sanatorium. I have had twenty months' general training.—*Nurse E.*

You can only obtain such employment by advertisement, or through private introductions.

*Maternity Home.*

(231) Will you kindly tell me how I could best obtain maternity cases in my own house? Would it be best to procure competent nurses, or should I qualify myself in monthly—not midwifery—nursing?—*E. A. D.*

We should recommend you to qualify yourself thoroughly in midwifery and obstetric nursing before venturing to receive patients. Then you will also know if the nurses you employ are competent.

*Masseuse.*

(232) Will you kindly tell me if there is a masseuse in Reading?—*M. L. B.*

Doubtless the Secretary, the Incorporated Society of Trained Masseuses, 12 Buckingham Street, Strand, W.C., would be able to give you the name and address of a good masseuse in Reading, or a local doctor might be able to help you.

*Sanitary Inspectorship.*

(233) What are the prospects of lady sanitary inspectors? Would a nurse holding the certificate of the Sanitary Institute, London, have a reasonable prospect of an appointment?—*A. M. F.*

Yes, as there is a growing demand for really competent lady inspectors.

*Infantile Paralysis.*

(234) Is there any children's home where a labourer's child, aged 4½, suffering from infantile paralysis of the legs, could be received for treatment? And is there any instrument by which he could be enabled to stand or walk?—*District Visitor.*

Write in the first instance to the Secretary of the National Hospital for the Paralysed and Epileptic, Queen's Square, Bloomsbury, W.C. If the case is unsuitable for treatment there, possibly the secretary will advise you for which of the children's hospitals he is most eligible.

*Egypt. Canada.*

(235) Please give me the address of nursing institutions in Egypt; and also say if there is any private, or district, nursing institution in Canada through which a nurse could obtain work; or, if she would be likely to find work if she went out and set up for herself.—*G. E. C.*

There is a private Nursing Home at Cairo, and there are several hospitals employing English nurses at the same place. See "Burdett's Hospitals and Charities" for the latter. English nurses are also employed at the Deaconesses' Hospital, Alexandria, and at Lady Strangford Hospital, Port Said. The Victorian Order of Nurses for Canada, 578 Somerset Street, Ottawa, is the most likely medium for securing district work in that colony. There is always the greatest risk in going abroad without previous introductions and prospects.

**Important Nursing Textbooks.**

"The Nursing Profession: How and where to Train." 2s. net; 2s. 4d. post free.

"A Handbook for Nurses." (New Edition). 5s. net; 5s. 4d. post free.

"The Human Body." 5s. post free.

"Ophthalmic Nursing." (New Edition). 3s. 6d. net; 3s. 10d. post free.

"Gynæcological Nursing." 1s. post free.

"Art of Feeding the Invalid." (Popular Edition). 1s. 6d. post free.

"Practical Hints on District Nursing." 1s. post free.

